# *Arthrospira maxima* and biosynthesized zinc oxide nanoparticles as antibacterials against carbapenem-resistant *Klebsiella pneumoniae* and *Acinetobacter baumannii*: a review article

**DOI:** 10.1186/s12934-024-02584-x

**Published:** 2024-11-19

**Authors:** Mohamed I. Selim, Tarek El‑banna, Fatma Sonbol, Engy Elekhnawy

**Affiliations:** Pharmaceutical Microbiology Department, Faculty of Pharmacy, Tanta University, Tanta, 31527 Egypt

**Keywords:** *Klebsiella pneumoniae*, *Acinetobacter baumannii*, Carbapenem-resistance, Spirulina, Algae, *Arthrospira maxima*, ZnO-NPs

## Abstract

Carbapenem resistance among bacteria, especially *Klebsiella pneumoniae* and *Acinetobacter baumannii*, constitutes a dreadful threat to public health all over the world that requires developing new medications urgently. Carbapenem resistance emerges as a serious problem as this class is used as a last-line option to clear the multidrug-resistant bacteria. *Arthrospira maxima* (Spirulina) is a well-known cyanobacterium used as a food supplement as it is rich in protein, essential minerals and vitamins and previous studies showed it may have some antimicrobial activity against different organisms. Biosynthesized (green) zinc oxide nanoparticles have been investigated by several researchers as antibacterials because of their safety in health. In this article, previous studies were analyzed to get to a conclusion about their activity as antibacterials.

## Introduction

Antibiotic resistance puts the health of the general public in considerable danger and makes treating it a challenge for medical professionals [1, 2]. Superbugs, which are multidrug-resistant microorganisms, are nearly resistant to most antibiotics available in the market which constitutes a serious burden that pharmaceutical companies struggle to cope with because of its high rate of spread and the slow processes of new medications discovery [3–5]. The intestinal bacteria *Klebsiella pneumoniae* is a Gram-negative, non-motile, opportunistic pathogen [6] that can infect other tissues and is a typical component of the microbiome found in healthy persons on the mucosal surfaces of the gastrointestinal tract and oropharynx. In hospitals and clinics, it results in a range of dangerous infections, especially in immunocompromised individuals, infants, and other high-risk patients. These infections include pneumoniae, meningitis, septicemia, and purulent abscesses [7]. Typically, it results in bloodstream infections, urinary tract infections (UTIs), pneumonia, and other hospital-acquired illnesses [8]. The irrelevant overuse of antibiotics has resulted in repeated outbreaks of multidrug-resistant *K. pneumoniae* [9].

One of *Acinetobacter baumannii*’s most well-known traits is its extreme antibiotic resistance. *A. baumannii* is a rod-shaped, aerobic, non-motile, Gram-negative coccobacillus that primarily causes illnesses that are potentially fatal and are obtained in hospitals. Immunocompromised patients especially those who had stayed for so long in the hospital are more prone to these infections with a high incidence [10, 11]. In recent years, it has become a growing burden to humans because of the widely distributed spectrum of antibiotic resistance [12]. It accounts for over 2% of all infections that are identified in hospitals in the United States. However, infection rates in critical care units (ICUs) are as high as 20% worldwide, with rates doubling in Asia and the Middle East [13, 14]. One of the main factors contributing to the pathogenicity of *Acinetobacter* is its ability to survive for extended periods on dry surfaces, as it makes it easier for the bacteria to spread across polluted hospital environments [15].

A class of medicines known as carbapenems is frequently used to treat these multidrug-resistant illnesses. Nevertheless, overuse or abuse of these antibiotics caused the spread of bacteria resistant to them, worsening the issue of antibiotic resistance and leaving us with less viable treatment options. The generation of carbapenemase, the efflux pump, and changes to porins are the mechanisms underlying carbapenem resistance. However, the primary and crucial process is the synthesis of the carbapenemase enzyme mediated by plasmids [16]. The genes responsible for producing carbapenemase are *bla*-*K. pneumoniae* carbapenemase (*bla*_KPC_), *bla*-oxacillin hydrolyzing enzymes-48 (*bla*_OXA−48_), *bla*-New Delhi metallo-β-lactamase (*bla*_NDM_), *bla*-Verona integron-mediated metallo-β-lactamase (*bla*_VIM_), and *bla*-active on imipenem (*bla*_IMP_) [17].

The global demand to develop new drugs is urgently needed to face this burden. Among the approaches is the use of natural sources like green algae as antibacterials [18]. *Arthrospira maxima* (spirulina) is a blue-green algae (cyanobacterium) that has gathered commercial interest. In recent years, its benefits as a nutraceutical have gained increasing attention because of its high protein content. It has been widely used as a food supplement as it contains all necessary amino acids, essential fatty acids, minerals, pigments, carotenoids, and vitamins [19]. It has also demonstrated antioxidant, antibacterial [20], antiviral [21], antifungal potential [22], and immune enhancer.

Biosynthesized zinc oxide nanoparticles (ZnO-NPs) have been studied against bacteria due to their safety and low toxicity compared to chemically synthesized ones [23, 24]. The biosynthesized ZnO-NPs have shown antibacterial action against a range of microorganisms, including Gram-positive and Gram-negative [25].

## The *Enterobacteriaceae* family

### Characteristics of *Enterobacteriaceae*

*Enterobacteriaceae* is a diverse, Gram-negative bacterial community that can thrive in different environments, including the intestines of humans and animals, soil, and water. These bacteria are distinguished by their rod shape, facultative anaerobic metabolism, and the ability to ferment glucose. They also have certain mutual antigenic properties, such as the presence of the O, H, and K antigens, which are important for serotyping [26].

Numerous well-known genera, including *Salmonella*, *Escherichia*, *Shigella*, *Klebsiella*, *Enterobacter*, and *Proteus*, are members of this family. This family has considerable medical concerns due to their involvement in illnesses linked to healthcare and the community, as well as in the context of antibiotic resistance [27]. Because of their ongoing evolution, *Enterobacteriaceae* have the potential to naturally duplicate themselves. They do this by developing resistance to the currently available antimicrobial agents, which makes the antibacterial agents ineffective, and by developing resistance to carbapenems, a last-line antibiotic option [28].

*Enterobacteriaceae*’s family members are normally distinguished by their rod shape and facultative anaerobic metabolism which means they can grow with or without oxygen, allowing them to adapt to various environments. This metabolic inconstancy is one of the main factors that contribute to their widespread presence and adaptability [29].

### Habitat and distribution

The gastrointestinal systems of both people and animals, as well as soil and water, are home to the widespread *Enterobacteriaceae* family. In the intestines, their existence aids digestion and the synthesis of essential vitamins. However, many are pathogenic and may cause several diseases including gastrointestinal infections, urinary tract infections, and septicemia [30].

### Prevalence of *Enterobacteriaceae*

*K. pneumoniae*, *E. coli*, *Proteus*, and *Enterobacter* spp. are often isolated more frequently from different clinical specimens than other *Enterobacteriaceae* spp [31]. Along with additional opportunistic infections (*Citrobacter* and *Klebsiella* spp.) in various clinical cases [32], *K. pneumoniae*,* Enterobacter* spp., *E. coli*, and *Citrobacter* spp. were causatives for some gastrointestinal diseases such as gastroenteritis, cholera-like syndrome, food poisoning, diarrhea, in addition to other diseases such as cystitis, pyelonephritis, appendicitis, pyelitis, peritonitis, lobar pneumonia, and septicemia [33]. Furthermore, *K. pneumoniae* is a common cause of nosocomial infections and mostly can cause pneumonia, urinary tract, central nervous system, hepatic, wound, and/or blood infections and is also associated with intestinal infections [34].

## *Klebsiella pneumoniae* as Gram-negative bacterium

### Characteristics of *K. pneumoniae*

In 1882, Carl Friedlander became the first person to describe *K. pneumoniae*. He identified the bacteria as an encapsulated bacillus after it was extracted from the lungs of pneumonia victims. It was once known as Friedlander’s *Bacillus* until 1886 when it was renamed *Klebsiella*. *K. pneumoniae* is a Gram-negative, naturally occurring, encapsulated, non-motile bacteria that has been connected to pneumonia in people with alcohol use disorders or diabetes mellitus. In humans, it typically colonizes the mucosal surfaces of the oropharynx and gastrointestinal (GI) tract. As soon as it enters the body, it demonstrates significant levels of pathogenicity and antibiotic resistance. Currently, 3–8% of all nosocomial bacterial infections in the US are hospital-acquired, with pneumonia due to *K. pneumoniae* being the most common cause [35–37].

### Etiology of *K. pneumoniae*

*K. pneumoniae* belongs to the *Enterobacteriaceae* family of bacteria and is an encapsulated, non-motile, Gram-negative bacterium. Multiple variables which can result in infection and drug resistance contribute to its pathogenicity. The most important virulence component is the polysaccharide capsule, which shields the bacteria from opsonophagocytosis and serum killing by the host organism. The second element of pathogenicity is the lipopolysaccharides that coat the outer surface of Gram-negative bacteria. Sepsis and septic shock are caused by an inflammatory cascade that is unleashed in the host organism upon detection of the lipopolysaccharides. Fimbriae, a further virulence factor, allows the bacteria to adhere to host cells. Another virulence component required by the organism to infect hosts is a siderophore. Siderophores take up iron from the host, which helps the infected organism to spread [38, 39].

### Epidemiology of *K. pneumoniae*

Humans are the primary reservoir for *K. pneumoniae.* It is estimated that in the community, 5–38% of people are carriers in the stool and 1–6% of them are carriers in the nasopharynx. The major sites are the GIT and hands, and it can lead to a nosocomial outbreak. Hospitalized patients had a much higher carrier rate of *K. pneumoniae* than the general population. A study found that 77% of stool samples from hospitalized patients had carrier rates, and this was correlated with the number of antibiotic prescriptions they had [40, 41].

Pneumonia caused by *K. pneumoniae* can be classified as either community- or hospital-acquired. Community-acquired pneumonia is a common diagnosis, while *K. pneumoniae* infection is uncommon. *K. pneumoniae* infections cause 3–5% of community-acquired pneumonia cases in wealthy countries like the United States; in developing countries like Africa, on the other hand, they cause about 15% of cases. *K. pneumoniae* accounts for 11.8% of hospital-acquired pneumonia cases globally. *K. pneumoniae* is the cause of 8–12% of pneumonia cases in patients using a ventilator, as opposed to 7% in people not using one. Alcoholism and septicemia both have 50–100% fatality rates [42].

### Pathogenesis and human infections caused by *K. pneumoniae*

*K. pneumoniae* is a common cause of a lot of infections in humans that range from local to systemic and from mild to acute or life-threatening infections. It’s an opportunistic pathogen that can cause a range of diseases in different parts of the body. The common opportunistic *K. pneumoniae* infection primarily targets immuno-compromised individuals or those with reduced immunity from prior infections. Nonetheless, hypervirulent *K. pneumoniae* is highly invasive and can cause severe pneumonia, meningitis, necrotizing fasciitis, endophthalmitis, and pyogenic liver abscess in healthy individuals. These are only a few of the community-acquired diseases that it can cause [43]. Hospital-acquired infections typically begin with *K. pneumoniae* colonizing the GIT. Furthermore, the bloodstream, respiratory system, and urinary tract may all become colonized by this infection [34].

#### Pneumonia

*K. pneumoniae* is widely recognized by most medical professionals as the cause of community-acquired bacterial pneumonia, which mostly affects chronic drinkers [44] and exhibits typical radiographic abnormalities brought on by a serious pyogenic infection, which, if left untreated, has a high death rate [45]. Nonetheless, the vast majority of *Klebsiella* infections are associated with hospitalization.

Because *Klebsiella* species are opportunistic, they usually target hospitalized patients with immune system compromises who have significant underlying medical problems such as diabetes mellitus or prolonged airway blockage. Nosocomial *Klebsiella* infections are mostly caused by *K. pneumoniae*, the genus’ most important species from a medicinal standpoint [34]. Although it can also infect the lower lobes, *K. pneumonia* often affects the upper lobes. Unilateral consolidation symptoms, particularly in the upper lobe, such as enhanced voice resonance, bronchial breathing, and crepitation, are typically seen on examination. It is important to look for burns, wounds, and intrusive equipment in cases of nosocomial infections.

#### Urinary tract infections

*K. pneumoniae* has been widely associated with nosocomial catheter-associated UTIs, where the catheter and percutaneous nephrostomy tubes are usually the colonization sites of this pathogen. Particularly in hospital settings, *K. pneumoniae*-related UTIs seem to be becoming more common and are now considered a serious health concern. Since infections are the second greatest cause of death in patients with chronic kidney disease (CKD), after cardiovascular issues, urinary infections might accelerate the course of renal failure in these patients. As a result, more caution should be used when handling this [46, 47].

#### Bloodstream infections

The second most frequent cause of Gram-negative bloodstream infections (BSI) that are acquired in hospitals and the community, after *E. coli*, is *K. pneumoniae* [34]. It usually results from a known source that enters the bloodstream and spreads. Intravenous or urinary catheters, the gastrointestinal tract, the respiratory system, and the urinary tract are common sources of secondary bloodstream infections (BSI) [48]. Death rates from *K. pneumoniae*-related BSIs are significantly elevated globally. ICUs and emergency departments had the greatest rates of bloodstream infections caused by *K. pneumoniae* [49, 50].

#### Meningitis

Less than 3% of instances of community-acquired bacterial meningitis are caused by *K. pneumoniae* [51]. On the other hand, data show that individuals with *K. pneumoniae* infection had a 48.5–66.0% mortality rate. In adult patients, it represents 50% of instances of severe meningitis [52] and is also associated with brain abscesses and endophthalmitis [53, 54]. Clinical outcomes for *Klebsiella pneumoniae* meningitis have been poor as similar cases have led to disseminated intravascular coagulation (DIC) and even recurrent infection [55].

#### Wound infections

As one of the main nosocomial pathogens, *K. pneumoniae* can infect human skin and mucosae, leading to severe local and systemic illnesses from wound infections [56]. The prognosis of wound infections is typically made more difficult by MDR *K. pneumoniae*, a feature that emphasizes the urgent need for novel therapeutic alternatives against these bacterial pathogens [57].

#### Liver abscess

Pyogenic liver abscess (PLA), caused by *K. pneumoniae*, is presently widespread worldwide. It can result in catastrophic intra-abdominal infections in septic persons. It is a frequent and potentially fatal consequence of a *K. pneumoniae* infection that can be made worse by metastatic endophthalmitis and central nervous system involvement, which can result in purulent meningitis or a brain abscess [58]. In both Asia and the USA, *K. pneumoniae* is the most frequently occurring isolate that causes PLA, accounting for over 60% of cases of both monomicrobial and polymicrobial PLA infections [59, 60].

### Hypervirulent (hypermucoviscous) *K. pneumoniae*

*K. pneumoniae* possesses a novel hypervirulent (hypermucoviscous) variety. First reported in the Asia-Pacific Rim, it is now becoming more widely acknowledged in Western countries. The ability of enteric Gram-negative bacilli to spread metastatically in non-immunocompromised hosts, as well as the unusual ability to induce severe, potentially fatal community-acquired infections in younger, healthy hosts, such as liver abscesses, pneumonia, meningitis, and endophthalmitis, are defining clinical features. Even with a healthy population, there is a considerable rate of illness and mortality. The path of entrance is yet unknown, but colonization, especially intestinal colonization, seems to be a crucial step before infection, even though epidemiologic characteristics are still being determined [43].

### Virulence factors of *K. pneumoniae*

**Fig. 1:**
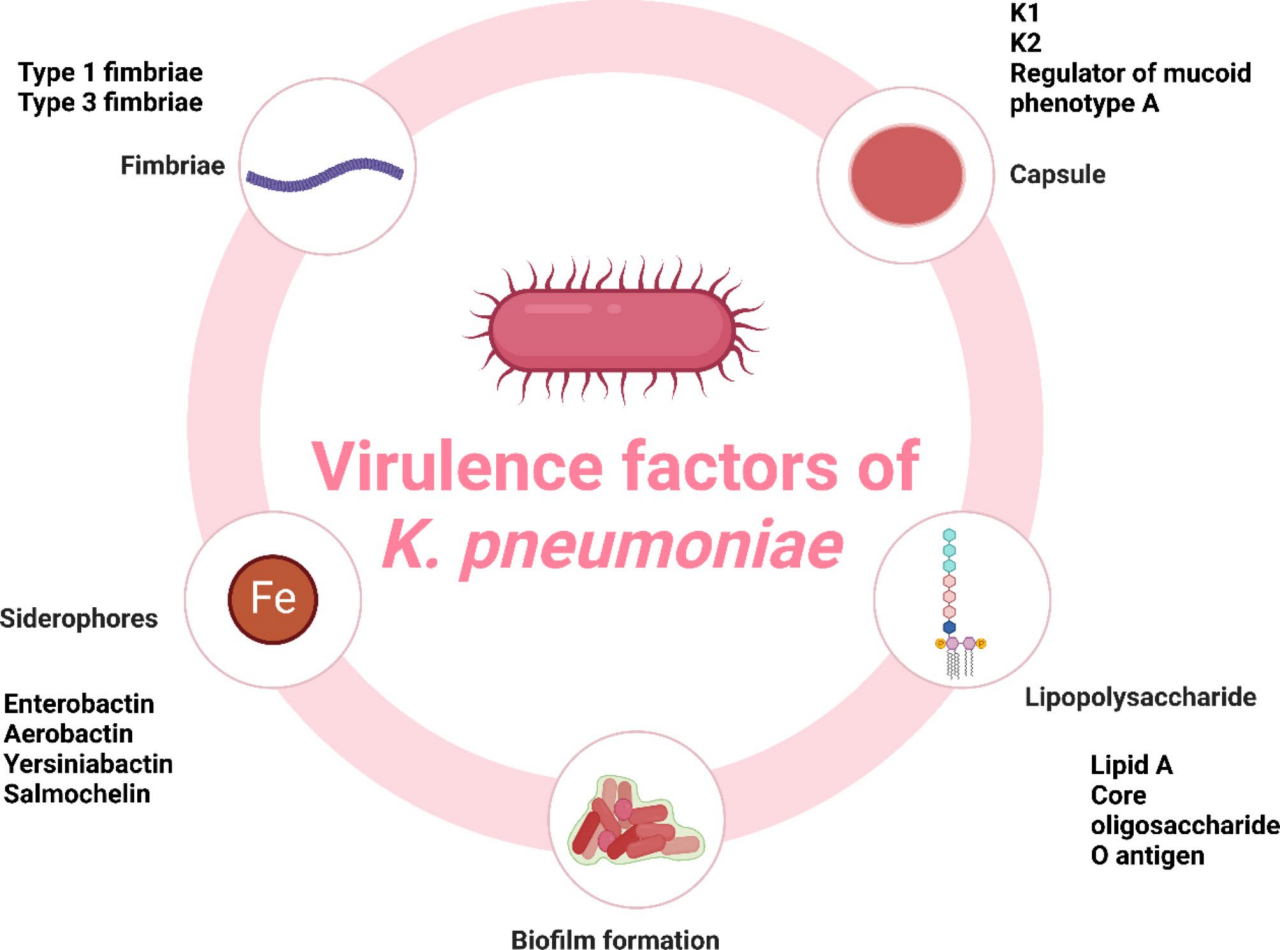
Virulence factors of *K. pneumoniae*

The definition of microorganism virulence is its ability to attach itself to a potential host, to attack and reproduce within that host, which results in local and/or systemic disease, and eventually to devastate and even eradicate the host [61]. This Gram-negative bacterium is renowned for its extensive virulence factors which enhance its virulence. Comprehending these virulence variables is essential to formulating efficacious treatment approaches and overseeing *K. pneumoniae* infections (Fig. 1).

#### The bacterial capsule

One of *K. pneumoniae*’s most important virulence agents is the polysaccharide capsule. It’s a dense, gel-like layer that surrounds the bacterial cell, providing protection against various host defense mechanisms. The capsule is important because it prevents complement from destroying bacterial cells and shields the bacterium from being phagocytosed by host immune cells. Strains of *K. pneumoniae* that produce a thick, mucoid capsule are more virulent compared to non-capsulated strains. The antiphagocytic properties of capsule are primarily due to its ability to mask surface antigens, thus preventing recognition by the host immune system [34].

Many capsular serotypes can be produced by *K. pneumoniae*, with K1 and K2 being especially linked to hypervirulent strains. Severe infections, such as liver abscesses and metastatic infections, are associated with these serotypes. The capsular polysaccharide synthesis (CPS) gene cluster contains the genes involved in capsule production [62]. The capsule also helps to increase resistance to serum’s bactericidal effects. Serum contains complement proteins that can form membrane attack complexes, leading to bacterial lysis. These complement proteins cannot attach to the bacterial surface because of the capsule, thereby enhancing serum resistance [63].

#### Lipopolysaccharide (LPS)

Lipopolysaccharide (LPS) is a major component of the outer membrane of Gram-negative bacteria, including *K. pneumoniae*. LPS is made up of three parts: O-antigen, core oligosaccharide, and lipid A. Each component of them has a unique function in the pathogenicity of the bacteria and how they interact with the host immune system.

##### Lipid A

Lipid A is the hydrophobic anchor of LPS, embedded in the bacterial outer membrane. It is the primary endotoxic component of LPS, accountable for inducing in the host a strong immunological response. Lipid A binds to Toll-like receptor 4 (TLR4) on immune cells when it is detected by the host’s immune system, activating inflammatory signaling pathways. Pro-inflammatory cytokines are produced as a result, which can lead to fever, inflammation, and, in extreme situations, septic shock [64].

##### Core oligosaccharide

The core oligosaccharide connects lipid A to the O-antigen. It consists of an inner core and an outer core. The inner core is more conserved among different strains of *K. pneumoniae*, while the outer core varies. The core oligosaccharide contributes to the structural integrity of LPS and plays a role in resistance to host antimicrobial peptides.

##### O-antigen

The O-antigen is the polysaccharide portion of LPS that extends out of the bacterial cell. It is highly variable and consists of repeating sugar units. This fluctuation hinders the establishment of a focused immunological response, which aids the bacterium in evading the host immune system. The O-antigen also provides resistance to complement-mediated lysis, as it can interfere with the binding of complement proteins [65].

#### Adhesins

On the surface of *K. pneumoniae* are hair-like projections called adhesins, such as pili and fimbriae, which are essential for adhering to host cells and surfaces and starting infections. Numerous adhesin subtypes have been found, and each one plays a different role in the pathogenicity and colonization of bacteria.

##### Type 1 fimbriae

The hair-like projections known as type 1 fimbriae facilitate *K. pneumoniae*’s attachment to mannose residues on the surface of host cells. Because type 1 fimbriae aids in the colonization of the urinary tract epithelium, this adhesion is especially crucial in cases of urinary tract infections [66].

##### Type 3 fimbriae

Type 3 fimbriae contribute to the development of biofilms and cling to abiotic surfaces, like those seen on medical equipment. It facilitates the binding of extracellular matrix proteins, such as fibrinogen and collagen. The production of biofilms, which are organized bacterial populations that are extremely resistant to drugs and immunological reactions, depends on this binding [67].

Apart from type 3 and type 1 fimbriae, type IV pili aid in the pathogen’s adhesion to epithelial cells, thereby promoting colonization and infection. They also play a role in the formation of biofilms, which further perpetuates infections, particularly on indwelling medical devices such as ventilators and catheters [68].

#### The role of siderophores

Microorganisms such as bacteria secrete siderophores, which are tiny, highly-affinity molecules that help them sequester iron from the surrounding environment. Iron is a critical nutrient for bacterial growth and metabolism, but it is typically limited in bioavailability within the host due to its sequestration by host proteins such as transferrin and lactoferrin. *K. pneumoniae*, like many other pathogenic bacteria, has evolved to produce various siderophores to overcome this limitation and flourish under conditions limited by iron, like those that exist inside the host when the infection is occurring.

##### Types of siderophores in *K. pneumoniae*

*K. pneumoniae* produces several types of siderophores, each with distinct properties and roles in iron acquisition and pathogenicity. The siderophores that *K. pneumoniae* produces that are the most understood include salmochelin, enterobactin, aerobactin, and yersiniabactin.

##### Enterobactin

The principal siderophore produced by *K. pneumoniae* is enterobactin, often referred to as enterochelin, and it exhibits a very high affinity for ferric iron (Fe^+ 3^).

##### Aerobactin

Aerobactin is another important siderophore produced by certain strains of *K. pneumoniae*, particularly those associated with hypervirulence. Unlike enterobactin, aerobactin has a slightly lower affinity for iron but compensates by being produced in larger quantities.

##### Yersiniabactin

Yersiniabactin is particularly important for the establishment of respiratory tract infections and sepsis. It helps *K. pneumoniae* overcome iron limitation in the host and contributes to immune evasion by scavenging iron from host proteins such as transferrin and lactoferrin [69].

##### Salmochelin

Salmochelin is an enterobactin derivative that has undergone glycosylation. This modification provides an advantage in evading the host’s immune defenses. Table 1 reveals the role of siderophores in *K. pneumoniae* infection.

**Table 1:** Role of siderophores in *K. pneumoniae*’s infection

Name of siderophore	Role of the siderophore
**Enterobactin**	Acquisition of required iron
**Aerobactin**	Associated with hypervirulence and acquires necessary iron
**Yersiniabactin**	Establishment of respiratory tract infections and sepsis
**Salmochelin**	Evasion of host’s immune defenses

#### Biofilm formation in *K. pneumoniae*

Biofilm formation is a crucial virulence factor in *K. pneumoniae*, facilitating its persistence in both medical and natural environments [70]. A biofilm is an organized colony of bacteria contained in an extracellular matrix that the bacteria manufacture on their own. Biofilms stick to surfaces like environmental substrates, human tissues, and medical equipment. This matrix is composed of extracellular polymeric substances (EPS), which include proteins, polysaccharides, and nucleic acids. EPS provides structural stability and protection. Numerous adhesins are employed by *K. pneumoniae* to promote and sustain the production of biofilms. Of special significance are, type 1 and type 3 fimbriae [66, 67, 71].

Because of the biofilm’s slow-growing or inactive cells, which are less susceptible to antibiotics, and its strong extracellular matrix, which restricts the penetration of antimicrobial drugs, biofilms are naturally resistant to both immunological responses and antibiotics. This resistance complicates treatment and often necessitates prolonged antibiotic therapy or the removal of infected devices. Particularly dangerous biofilms can result in prolonged infections and higher morbidity on medical devices like ventilators and urine catheters [72, 73].

### Treatment options for *K. pneumoniae*

*K. pneumoniae* antimicrobial resistance has been a major concern in published recent studies, especially in the existence and the rising problem of multidrug-resistant strains, extensively drug-resistant strains, which are resistant to all antibiotics except for one or two classes, even pan-drug-resistant strains. This problem has a great interest among many hospitals in the world, as it increases the mortality rate, hospital stay length increased by two times, and healthcare costs increased too. Treatment options for *K. pneumoniae* infections depend on factors such as the infection’s type, severity, and the bacteria’s antibiotic susceptibility. Here are the key treatment strategies:

1. **Antibiotics**: Antibiotics are the primary treatment for *K. pneumoniae* infections. Choices typically include carbapenems, fluoroquinolones, aminoglycosides, and third-generation cephalosporins. However, for multidrug-resistant strains, alternative antibiotics such as colistin and tigecycline, or combination therapies, may be necessary [74].
2. **Combination therapy**: For severe or complicated infections, combination antibiotic therapy can be more effective and help prevent resistance development. This approach uses multiple antibiotics with different mechanisms of action to target the bacteria more effectively [75].
3. **Antimicrobial susceptibility testing (AST)**: It is essential to perform AST before starting treatment to determine which antibiotics are most effective against the *K. pneumoniae* strain. This ensures optimal treatment and helps minimize resistance [76].
4. **Supportive care**: In severe cases such as pneumonia or bloodstream infections, supportive care is essential. This may include respiratory support (e.g., supplemental oxygen or mechanical ventilation), intravenous fluids, and other therapies to maintain vital organ function and manage symptoms [77].

## *Acinetobacter baumannii* as a Gram-negative bacterium

### Characteristics of *A. Baumannii*

*Acinetobacter* is a highly diverse genus that was first isolated through the 1940s and 1960s, back then, it was taxonomically classified as *Herellea vaginicola* (now *A. baumannii*). It’s interesting to note that *A. baumannii*, the most common species, wasn’t officially nominated until 1986. However, it appears that infections due to *A. baumannii* initially emerged as a serious issue in the 1970s [78]. *A. baumannii* is a common Gram-negative coccobacillus that is aerobic, oxidase-negative, non-pigmented, and found in wet habitats such as sewage, fish farms, ocean, moist soil/mud, and vegetables. Seldom has *A. baumannii* been recognized as a typical skin colonizer [79]. *A. baumannii* is thought to be the most prevalent infectious member of the *Acinetobacter* genus [80, 81].

### Epidemiology of *A*,* baumannii*

*A. baumannii*, with its ability to resist almost all used antibiotics, represents the most dangerous and life-threatening category [81]. It has been determined that in America and Europe, *A. baumannii* is responsible for 1.8% of all illnesses linked to healthcare, with even higher percentages in other countries such as China, India, Thailand, Taiwan, and Vietnam, where it may be considered a predominant nosocomial pathogen. Unfortunately, this superbug usually causes about 1 million cases globally per year [79]. The main risk factor for *A. baumannii* has been determined to be an extended stay in the intensive care unit. The prior use of antibiotic medication, mechanical ventilation, indwelling catheters, endotracheal tubes, nasogastric tubes, complete parenteral nutrition, intravenous lipids, and endotracheal tubes are additional risk factors. People with diabetes mellitus, long-term obstructive lung disease, significant burns or trauma, frequent smoking, and alcohol intake are also thought to be very susceptible to contracting the infection [82].

The World Health Organization (WHO) reports that *A. baumannii* resistant to carbapenems, is the top infection that needs new treatments immediately. There is persistent concern that *A. baumannii* infections obtained in hospitals could soon turn fatal, which could result in higher rates of morbidity, death, medical expenses, and antibiotic use [27, 83].

### Pathogenesis and human infections of *A. Baumannii*

Due to their high rates of fluid exchange, the peritoneal system, respiratory tract, and urinary tract are some of the organs and organ systems most impacted by *A. baumannii*. As a result, *A. baumannii* is regarded as the most frequent primary cause of bacteremia and nosocomial pneumonia [84]. Yet, it’s also connected to several illnesses, including UTIs, traumatic or surgical burn and wound infections, osteomyelitis, and post-neurosurgical meningitis [79]. Nonetheless, warm, humid, tropical climates like those found in Asia, China, Taiwan, and Thailand are the main places where community-acquired diseases happen. Meningitis, cellulitis, and primary bacteremia are uncommon symptoms of community-acquired *A. baumannii* infection; instead, acute pneumonia is thought to be the most common major symptom. In both nosocomial and community-acquired cases, inappropriate initial antimicrobials strongly worsen the infection and can even cause death [85].

#### Pneumonia

*A. baumannii* which is acquired in a hospital is widely regarded as the most frequent clinical symptom of *A. baumannii*. In hospitals, the risk of ventilator-associated pneumonia is greatly increased by prolonged stays in the intensive care unit, prolonged mechanical ventilation, and inappropriate early use of antibiotics. Regrettably, hospital-acquired pneumonia caused by *A. baumannii* can result in a 72% mortality rate for hospitalized patients who contract the infection [86].

In tropical and subtropical regions, community-acquired pneumonia has also been reported, though much less frequently. Excessive alcohol use is a primary risk factor for illness. About 64% of instances that have been reported have had a severe and quick start of pneumonia, which is followed by secondary bacteremia and ultimately results in mortality [87].

#### Bloodstream infections

*A. baumannii* has also been described as a frequent etiological pathogen implicated in bacteremia in intensive care settings. Lower respiratory tract infections, intravascular devices, wound infections, and UTIs are typically thought to be the areas where bloodstream infections occur most frequently. *A. baumannii* infection-related crude death rates in patients, which have reached as high as 56%, have been associated with BSIs. The only infections that exceeded the crude fatality rates from MDR *A. baumannii* infections were *P. aeruginosa* and *Candida* spp [88]. Furthermore, sepsis and septic shock brought on by MDR *A. baumannii* have been linked to extremely high death rates (82.5%) as a consequence of a deadly chain of events that is unlikely to be stopped, not even by a suitable course of early antimicrobial therapy [89].

#### Burn and wound infections

*A. baumannii* is a well-documented pathogen in burn units, and in cases of hospitalized severely wounded patients, even though, their prevalence considerably varies depending on the institution and its geographic location. Burn and wound infections are regarded as serious problematic infections because they can cause skin graft failure, a delay in wound healing, colonization of the wound site leading to soft tissue infection, and ultimately bacteremia [90].

#### Meningitis

*A. baumannii*-caused nosocomial post-neurosurgical meningitis has grown to be a significant issue in post-operative care, as the implantation of an external ventricular drain may act as a haven for opportunistic infections. However, only minor cases were reported concerning community-acquired meningitis caused by *A. baumannii*. This type of meningitis shows the usual clinical picture with fever, altered consciousness, headache, seizures, and eventually death [91].

#### Osteomyelitis

*A. baumannii*-induced osteomyelitis has been recognized as a serious risk factor for military personnel experiencing stress due to war, particularly during US military deployments in Iraq and Afghanistan. In one investigation on *A. baumannii* osteomyelitis in injured soldiers, all 18 patients reported the need for immediate surgical debridement of necrotic bone, and three of them went on to develop bacteremia [92].

#### Urinary tract infections

*A. baumannii* is one of the most common probable causes of nosocomial catheter-associated UTIs. The significant degree of antibiotic resistance found in bacteria isolated from intensive care unit patients may have been caused by a longer catheterization stay and more frequent use of broad-spectrum antibiotics. This could allow MDR *A. baumannii* to enter the bloodstream and develop into potentially fatal bloodstream infections [93].

### Virulence factors of *A. Baumannii*

**Fig. 2:**
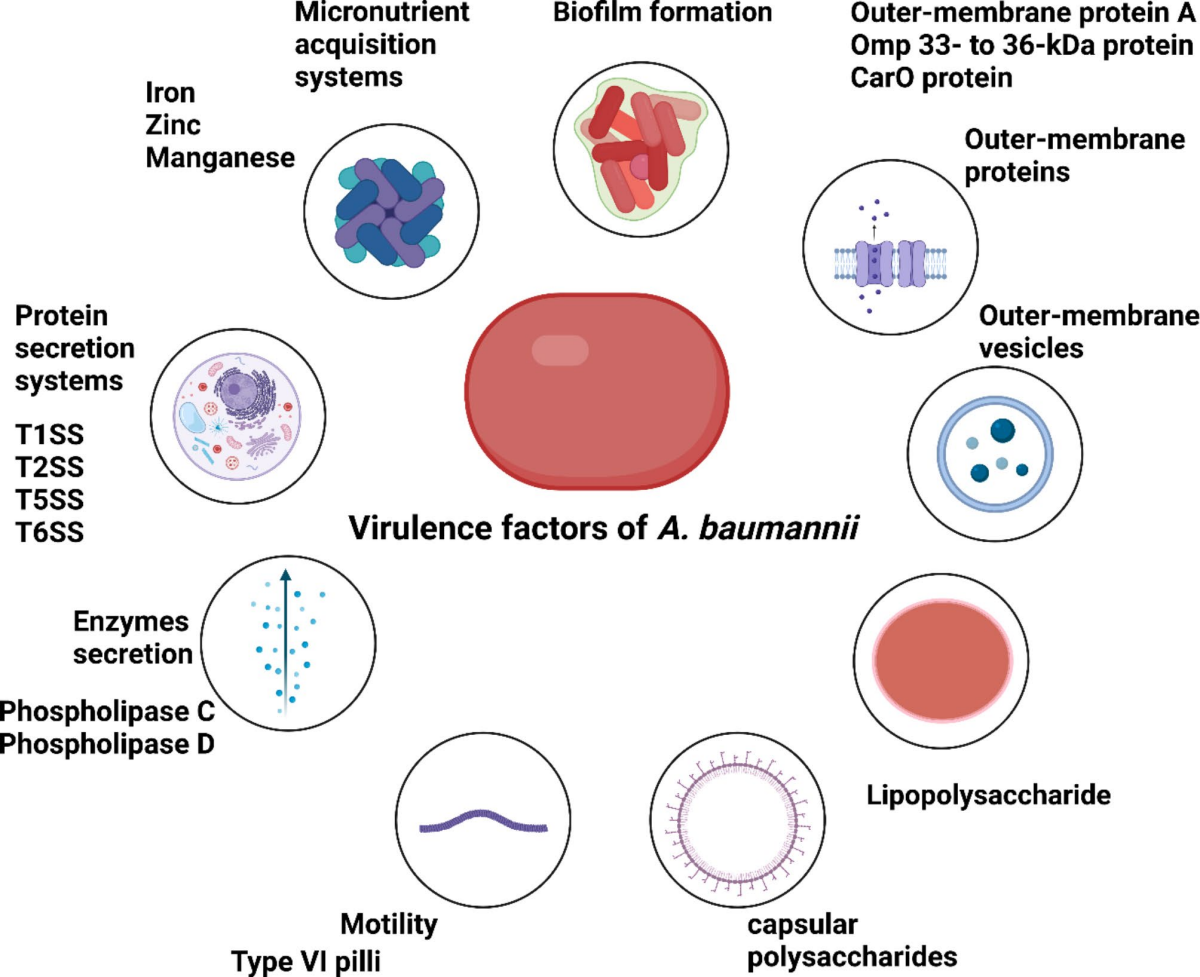
Virulence factors of *A. baumannii*

Researchers have worked very hard over the last decade to understand the unique and complex virulence factors of *A. baumannii*. Despite this, several characteristics of these bacteria have been found to differ from those of other Gram-negative microbes up until this point. An illustration of so far identified virulence factors of *A. baumannii* can be curtailed in Fig. 2 [82].

#### Outer-membrane proteins

On the surfaces of outer cells, outer-membrane vesicles (OMVs) carry outer-membrane proteins (OMPs), also known as porins. The most well-characterized virulence determinant in *A. baumannii* appears to be outer-membrane protein A (OmpA) [94]. It was discovered that *A. baumannii*’s OmpA membrane protein serves a variety of purposes (Nie et al., 2020). It has been found that OmpA increases the tenacity and survival of *A. baumannii* by encouraging surface motility and biofilm formation. Due to its crucial roles in the pathogenesis of *A. baumannii*, OmpA provided a very interesting candidate for novel therapeutics and vaccines [81]. The OMP 33–36 kDa protein, another outer-membrane protein of *A. baumannii*, serves as a water channel and its expression promotes the development of carbapenem resistance. CarO contributes significantly to the emergence of carbapenem resistance in *A. baumannii*, much as OMP 33–36 [95].

#### Outer-membrane vesicles (OMVs)

*A. baumannii*, secrete a major type of virulence factors called OMVs. OMVs are made up of phospholipids, LPS, and proteins found in the periplasm and outer membranes of bacteria. Via OMVs, *A. baumannii* can transfer several virulence factors into host cells without physical contact, including OmpA, putative hemolysin, phospholipases, and proteases. Furthermore, OMVs function as transporters that promote the development of antibiotic resistance in *A. baumannii* and facilitate horizontal gene transfer, such as the carbapenemase gene [81].

#### Lipopolysaccharides (LPS)

The cell wall LPS of *A. baumannii* is implicated in several stages of the progression of the disease, just like in all other Gram-negative human infections. LPS is composed of a repetitive O-antigen, a carbohydrate core, and a lipid A moiety. Whereas the antigenic O-polysaccharide of the LPS is thought to be a crucial component required for the pathogen’s attachment stage to the host cells. Additionally, it has been demonstrated that the detection of LPS by CD14 and Toll-like receptor 4 (TLR4) helps remove *A. baumannii* from the lung [96].

#### Capsular polysaccharides

Similar to other components linked to the cell envelope, such as LPS, capsules are essential to *A. baumannii*’s pathogenicity because they serve as a barrier against immune cells and harsh environmental elements like antibiotics and disinfectants. Additionally, *A. baumannii* was proven to survive under dry environmental conditions for a period that can reach 100 days, and that could be attributed to the capsular polysaccharides that can form a completely intact layer around each bacterial cell. LPS and LOS are first produced in the cytoplasm of the bacterial cell before being translocated, via a variety of proteinaceous methods that involve OMPs, to the cell envelope’s outer leaflet, where the bacterial cell builds its capsule. To produce the capsule, LPS and LOS are then used and packed together in various configurations.

#### Protein secretion systems

*A. baumannii* secretes several proteins through distinct secretory systems, which enable the pathogen to interact with host tissues and the environment and offer potential targets for treatment. As of right now, *A. baumannii* has been found to have many protein secretory systems, including type I, II, IV, V, and VI secretory systems [97].

The ATP binding transporter, the membrane fusion protein, and an outer membrane component that permits protein release through a single lipid membrane step comprise the three subunits of T1SS, which is present in *A. baumannii*. Substrates of T1SS vary according to the localization of the disease and are highly implicated in the biofilm formation step parallel to biofilm-associated protein (bap) expression [98].

T5SS is considered the simplest way of transportation as it does not necessitate any subsidiary elements or any other forms of energy. The three primary domains that comprise T5SS are the autotransporter domain, the passenger domain, and the signal sequence. It functions to mediate bacterial attachment to host tissue constituents, especially collagen, then proceeds in the virulence biofilm formation/maintenance process [99].

Furthermore, *A. baumannii* exports several effector proteins via a T2SS. Proteins are translocated via this system either the general secretory system or the twin arginine transport system to the periplasm, where they are then effluxed out of the cell. The metallopeptidase (glycan-specific adamalysin-like protease) and lipases (LipA and LipH) have been identified as T2SS substrates [97].

*A. baumannii* can introduce protein poisons into bacterial cells through the contact-dependent inhibition system (CDI) and T6SS, respectively, either in the same population (intra-species killing) or in other competing bacterial species (inter-species killing). To attack other bacteria, T6SS generates toxins including nucleases, peptidoglycan hydrolases, and cell-membrane active toxins. However, some clinical strains of *A. baumannii* have developed exquisite forms to regulate the function of T6SS. For example, a large conjugative plasmid including repressors of the T6SS and numerous antibiotic resistance genes, including tetracycline, is present in multiple strains. Thus, loss of plasmid renders T6SS active and produces potent bacterial killers of *A. baumannii*. However, these murderers now exhibit susceptibility to administered antibiotics, indicating a molecular transition between T6SS and antibiotic resistance [100].

#### Biofilm formation

One important virulence feature of *A. baumannii* that aids in its colonization of host-associated biotic surfaces, including collagen, is the creation of biofilms. Moreover, *A. baumannii* is a major cause of device-related infections due to its ability to attach to and colonize abiotic surfaces, such as wound dressings and indwelling medical devices. A collection of bacterial cells embedded in an extracellular polymeric material (EPS) that the cells themselves create is referred to as a biofilm [82].

#### Enzymes

A significant class of lipolytic enzymes known as bacterial phospholipases are connected to the pathophysiology and pathogenicity of numerous Gram-negative bacteria. Phospholipases act through cleavage and destruction of phospholipids located in the membranes of host cells leading to its destruction. Moreover, they act on phospholipids in the mucosal barriers of the host facilitating invasion of bacteria through the host tissues. *A. baumannii* has been shown to contain two phospholipases: phospholipase D (PLD) and phospholipase C (PLC). PLC cleaves the phosphorylated head group off the phospholipid, whereas PLD, a transphosphatidylase, only cleaves off the head group. The host cell membranes become less stable as a result of the breakdown of the phospholipids [101].

#### Motility

*Acinetobacter* has long been thought to be non-motile since its name comes from the Greek word “nonmotile,” a-kineto, and because *A. baumannii* does not have flagella. Nevertheless, it has been shown that motility is among the most likely virulence factors for this genus. Where, *A. baumannii* showed “twitching” motility on surfaces of agar plates and in response to illumination, quorum sensing, and iron chelation. Using type IV pili, this twitching motility is achieved by pushing cells in the medium through extension and retraction movements. Additionally, type IV pili are implicated in biofilm formation and gene transfer [102]. Some other strains of *A. baumannii* show another type of movement that occurs on animate and inanimate surfaces regardless of flagella. This type of motility is called surface-associated motility and also requires type IV pili. However, the exact mechanism of this type of motility continues to be a mystery up till now [103].

#### Micronutrient acquisition systems

Transition metals including iron, zinc, and manganese are thought to be crucial for *A. baumannii*’s nutrition and metabolism. Thus, hosts utilize a strategy called nutritional immunity to evolve several mechanisms sequestering these metals from the pathogen. *A. baumannii* primarily uses siderophores, which are high-affinity iron chelating molecules, to scavenge iron. *A. baumannii* can sequester zinc molecules, which function as structural cofactors for numerous proteins, through the use of a high-affinity zinc acquisition system. It is thought that a transporter connected to the natural resistance-associated macrophage protein (NRAMP) family facilitates manganese accumulation and growth in environments where metal availability is limited, though the exact mechanisms by which *A. baumannii* overcomes manganese deficits are still being investigated [100].

### Treatment options for *A. Baumannii*

*A. baumannii*-associated infections are among the hardest illnesses to manage and cure due to their propensity to become resistant to almost every kind of medication. In the case of susceptible *A. baumannii* infections, β-lactams and broad-spectrum cephalosporins (ceftazidime or cefepime) are usually considered effective antibacterial therapies to which bacterial cells briskly respond. Unfortunately, increased antibiotic resistance has drastically limited all therapeutic options against *A. baumannii*. According to numerous investigations conducted worldwide, 70–90% of *A. baumannii* isolates were shown to be multidrug resistant to three or more antibiotics of different classes, like fluoroquinolones, aminoglycosides, carbapenems, penicillins, cephalosporins, and polymyxins [104].

Carbapenems are the cornerstone of treatment for MDR *A. baumannii*; the most efficient and dependable carbapenems are meropenem, imipenem, and ertapenem. Polymyxins, including polymyxin E, are polycationic peptide antibiotics that are effective against Gram-negative bacteria, including *A. baumannii*, through the destruction of their outer membrane integrity. Polymyxin E, commonly referred to as colistin, is often used as a stand-alone medication or as an essential component of combination therapy to treat CRAB infections. However, because of the drug’s potentially fatal nephrotoxicity and neurotoxicity, very little colistin is used. To sum up, *A. baumannii* has been gradually displaying increasing rates of resistance to polymyxins and tigecycline, the last-line treatments. These recently discovered PDR *A. baumannii* strains do highlight the urgent necessity of employing different treatment approaches to manage them [83].

## Carbapenem resistance in *Enterobacteriaceae*

Globally, antimicrobial resistance is becoming a bigger issue. Multidrug-resistant organisms, or MDROs, are a global concern to public health and are spreading more widely. They have a connection to high rates of mortality and morbidity as well. In the past, MDROs have impacted patients in hospital environments where risks for acquisition include host characteristics, frequent and/or prolonged hospitalization, antibiotic exposure, and use of in-dwelling devices [105]. Treatment options to fight infection are limited due to *Enterobacteriaceae*’s growing resistance to antibiotics.

Carbapenems are widely used to treat infections caused by organisms resistant to antibiotics. Carbapenems are typically limited to MDR infections due to their extensive spectrum of action [106]. Carbapenems and penicillins share a similar structural makeup, but they also contain a pyrroline ring [107]. The penicillin-binding protein in the bacterial cell wall is broken down by both antibiotics using a beta-lactam ring; however, the addition of a pyrroline ring protects the beta-lactam ring from part of the bacterium’s degrading enzymes [107]. Serious implications arise for hospital epidemiology and individual patient treatment when *Enterobacteriaceae* evolve carbapenem resistance mechanisms [108].

Sadly, certain bacteria have evolved defense mechanisms against this family of drugs. We refer to these germs as *Enterobacterales* that are resistant to carbapenem (CRE). The presence of a carbapenemase or resistance to at least one carbapenem characterize CRE. Reduced expression of porin, efflux pumps, or the synthesis of carbapenemase are some of the processes that underlie resistance to carbapenem. The advent of carbapenem resistance emphasizes how important it is to develop new antibiotics and take excellent care of the ones that are presently in use [109].

The most prevalent CRE that produces carbapenemase is the *bla*_KPC_ gene [110]. The *bla*_KPC_ is considered endemic in several regions due to its emergence in bacterial populations, including the United States, Mexico, Portugal, Italy, and Greece [111]. *bla*_OXA−48_ carbapenemases have become more common throughout the Middle East, North Africa, and much of Europe, whereas *bla*_NDM_ carbapenemases are abundant in South Asia, the Mediterranean, Romania, Denmark, and Poland [77, 112, 113]. Whereas *bla*_IMP_ carbapenemases are mainly found in the Asia-Pacific region, which includes Australia, China, and Japan, *bla*_OXA−48_ carbapenemases are often found in Turkey [113]. The movement of populations across borders regularly can help certain carbapenemases spread to other areas.

### Carbapenem-resistant *K. pneumoniae*

Infections caused by isolates of carbapenem-resistant *K. pneumoniae* (CRKP) pose a major concern to public health. These infections may increase the mortality rates of very ill and incapacitated patients admitted to intensive care units (ICUs) and have a detrimental impact on the cost of those patients’ hospital stays on a worldwide scale [114, 115]. It is noteworthy that around 50% of people who get pneumonia due to *K. pneumoniae* die from the disease [116]. Another significant public health concern is the effect of CRKP infections on disability-adjusted life years (DALYs) per 100,000 population. Greece has one of the highest rates of disability-adjusted life years (DALYs) in the European Union (11.5) [117]. A recent meta-analysis found that whereas the prevalence of CRKP colonization varies globally from 0.13 to 22% with a pooled prevalence of 5.43%, the incidence of CRKP colonization ranges from 2 to 73% with a pooled incidence of 22.3% [118].

The designation of CRKP isolates as extensively drug-resistant (XDR), pan-drug-resistant (PDR), and multidrug-resistant (MDR) complicates the treatment of infections. The European Center for Disease Prevention and Control (ECDC) defines XDR as “non-susceptibility to at least one agent in all but ≤ two antimicrobial categories (i.e., bacterial isolates remain susceptible to only one or two categories)” and MDR as “acquired non-susceptibility to at least one agent in ≥ three antimicrobial categories”. In addition, PDR is defined as “non-susceptibility to all agents in all antimicrobial categories” [119]. The molecular epidemiology of CRKP isolates is important because it can be used to determine potential treatment strategies [120].

### Carbapenem-resistant *A. Baumannii*

Respiratory tract infections in critical care units (ICUs) are primarily linked to ventilator-associated pneumonia (VAP) by carbapenem-resistant *A. baumannii* (CRAB) [121, 122]. Although the association between CRAB infections and an increased mortality risk is not well understood, there is a strong association between CRAB infections and increased length of stay in the intensive care unit, increased patient costs, and antibiotic use [123]. For CRAB infections in intensive care units, polymyxin remains an effective treatment to this day [124]. However, due to nephrotoxicity and neurotoxicity, the usage of polymyxin in individual patients is still somewhat restricted [125]. There have also been reports of *A. baumannii* developing resistance to polymyxin [126]. Since these conditions may provide more challenges, the medical community has become more concerned about the management and treatment of CRAB in intensive care units.

### Mechanisms of carbapenem resistance

**Fig. 3:**
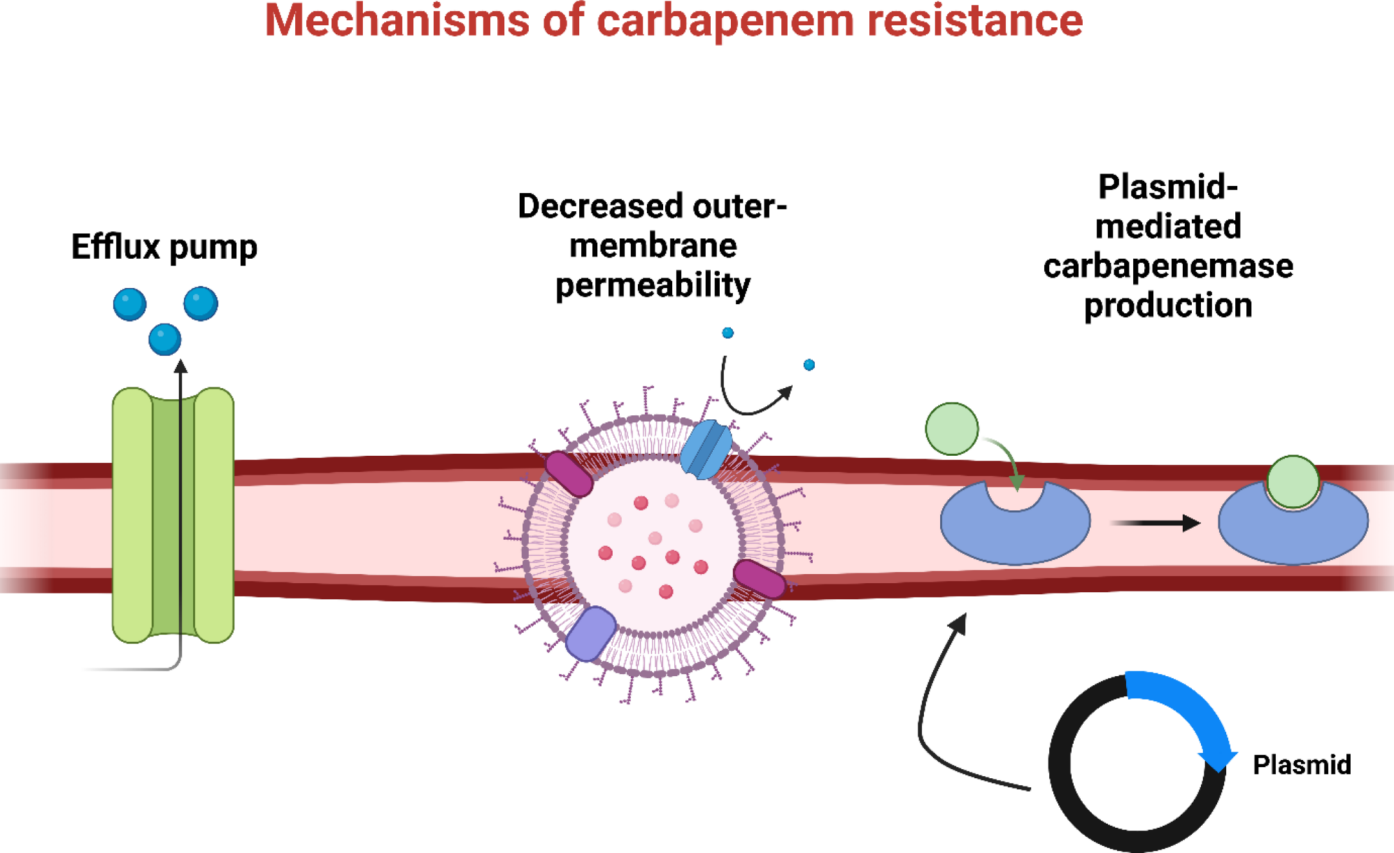
Mechanisms of carbapenem resistance

In certain species, the ability to withstand the effects of antibiotics through many routes accounts for their innate resistance to carbapenems. Apart from intrinsic resistance, bacteria can acquire antibiotic resistance by other processes, which can be classified into three primary classes. The first category includes antibiotics that do not sufficiently penetrate the outer membrane of the bacterium or exhibit antibiotic efflux. Bacteria in the second group modify the target of antibiotics by post-translational modifications or genetic alterations. To render carbapenems and other so-called carbapenemases inactive, the third class of bacteria develops beta-lactamases. This allows the bacteria to act by enzyme-catalyzed modification [127]. The mechanisms of carbapenem resistance are shown in Fig. 3.

#### Porin-mediated resistance

Bacteria can restrict carbapenem access into the periplasmic region, which is home to PBPs. Through modifications to porin expression or the porin-encoding gene, this process causes either a complete loss or deficiencies in the corresponding porin [128]. For example, the main mechanism of carbapenem resistance in *P. aeruginosa* isolates is the downregulation of the gene encoding the oprD porin [129]. Similarly, it was demonstrated that altered expression of ompk35 and ompk36 led to a significant degree of ertapenem resistance in *K. pneumoniae* [130].

#### Overproduction of efflux pumps

Efflux pumps can normally identify a wide range of substrates since affinity is based on physiochemical characteristics (such as electric charge, and aromatic or hydrophobic properties) rather than chemical structures. This explains why MDR efflux pumps can eject a wide variety of structurally unrelated antimicrobials [131]. It is commonly known that Gram-negative bacteria, such as *P. aeruginosa* and *Acinetobacter* species, are resistant to β-lactam antibiotics due to efflux-mediated mechanisms [132]. Overexpression of efflux pumps that are active on carbapenems may lead to carbapenem resistance [133].

#### Enzyme-mediated resistance

Resistance is typically caused by the creation of β-lactamases, or carbapenemases, which are capable of hydrolyzing carbapenems and other β-lactam antibiotics. This resistance mechanism poses the greatest threat because these enzymes can inactivate the majority of β-lactams and are encoded by genes carried on transposons, plasmids, or other mobile genetic elements that can be horizontally spread to other bacterial species [134].

Class A, class B, and class D are the three classes of β-lactamases into which carbapenemases are divided according to their molecular structures. Classes A and D are called serine β-lactamases (SBLs) because they have a serine residue at the active site that helps with ring opening [135]. The active site of Class B metallo-β-lactamases (MBLs) uses zinc ions to promote bond hydrolysis [132]. β-lactamase inhibitors, such as tazobactam, sulbactam, and clavulanic acid, can block SBLs. On the other hand, MBLs are unaffected by metal ion chelators, which inhibit them [136].

##### Class A carbapenemases

Class A carbapenemases include *K. pneumoniae* carbapenemases (KPCs), imipenem-hydrolyzing β-lactamase (IMI), Guiana extended spectrum carbapenemase (GES), and nonmetallo-carbapenemase-A [137]. The *bla*_KPC_ gene can hydrolyze any β-lactam antibiotic. Additionally, bacteria that carry this gene are usually multidrug-resistant, meaning they are resistant to trimethoprim-sulfamethoxazole, aminoglycosides, and fluoroquinolones, among other antimicrobials. The *bla*_KPC_ genes are plasmid-encoded, allowing for horizontal gene transfer between species [138].

Because of their distinct AMR profile, isolates that produce *bla*_IMI_ may be difficult to identify; these isolates typically exhibit imipenem resistance but only moderate ertapenem resistance and are vulnerable to extended-spectrum cephalosporins. Moreover, the *bla*_KPC_ gene is not included in the panel of genes that are the target of commercially accessible molecular diagnostic tests. *bla*_IMI−1_ carbapenemases are thought to be clinically unimportant because they are chromosomally encoded [139].

##### Class B carbapenemases

Plasmid-encoded imipenem-resistant Pseudomonas-type carbapenemases (*bla*_IMP_), which *P. aeruginosa* was reported to contain in 1991, sparked interest in this class of enzymes as a potential treatment [140]. These days, MBLs are primarily plasmid-encoded, which makes it easier for microbial pathogens to spread them [141]. They can also inactivate most β-lactams, save for monobactams, and are the class of carbapenemases with the most molecular variation [142]. The New Delhi MBL (*bla*_NDM_) is an MBL that can confer resistance to β-lactam antibiotics, particularly carbapenems, in enteric bacteria like *K. pneumoniae* and *E. coli* [143] although not aztreonam [144]. From an isolate of *P. aeruginosa* in Verona, Italy, the first description of Verona integron-encoded MBL (*bla*_VIM_) was published in 1999. Except for aztreonam, *bla*_VIM_’s hydrolytic profile hydrolyzes most β-lactams, just like other members of this class [141].

##### Class D carbapenemases

These include the enzymes called oxacillinase (OXA), which get their name from their ability to hydrolyze oxacillin efficiently [145]. The class D enzyme that was initially identified was *bla*_OXA−2_ β-lactamase [146]. The carbapenem-hydrolyzing *bla*_OXA−48_ enzyme exhibits poor hydrolysis activity toward carbapenems and high hydrolysis activity toward penicillins [147]. This enzyme has gained attention recently since β-lactamase inhibitors do not affect it either [148]. Although they are commonly found in Acinetobacter species, comparatively less carbapenemase activity is exhibited by other OXA β-lactamases, including *bla*_OXA−23_, *bla*_OXA−24/40_, and *bla*_OXA−58_ [149]. The absence of inhibitors for this class of enzymes is one of their biggest challenges [145].

## Non-antibiotic approaches to combat carbapenem-resistant infections

There is an immediate global demand to discover new medications to overcome these MDR infections. However, the process of developing new drugs and testing their safety, stability, and efficacy in humans is very slow and may take years. As a result, several researchers are testing many natural products and synthetic compounds for their activity as non-antibiotic approaches. Among these approaches, natural sources like green algae are tested as antibacterials. Thanks to its high protein content, all essential amino acids, vital fatty acids, minerals, pigments, carotenoids, and vitamins, Spirulina is a blue-green algae that is well-known in the food supplement industry [150]. Spirulina has also revealed antioxidant activity, immune enhancing activity, and antibacterial, antiviral, and antifungal potential. Green ZnO-NPs have been assessed by many researchers for their activity against bacteria and in the wound healing process because, in contrast to chemical ZnO-NPs, it is non-toxic and has little toxicity.

### *Arthrospira maxima*

*Arthrospira*, a cyanobacterium that was once known as Spirulina, grows quickly and is easy to cultivate. It generates a variety of chemicals used in biofuels, medicines, diagnostics, and natural dietary additives [151, 152]. *A. maxima* is a filamentous, unbranched cyanobacterium that contains vital cellular components like protein, essential fatty acids, vitamins, minerals, and iron [153]. Along with *Chlorella* and *A. platensis*, *A. maxima* is one of the most significant commercial photoautotrophs due to the rising demand for high-value phytonutrients and pigments worldwide [150, 154].

Naturally occurring in tropical lakes with high NaCl and bicarbonate waters (pH 11), spirulina can be found there. While other microorganisms cannot thrive in these conditions, spirulina can be grown in open reactors [154]. Almost anywhere can contain spirulina, including soil, brackish water, freshwater, ocean, marshes, and thermal springs. Lake Texcoco in Mexico and extremely alkaline lakes in Africa are ideal habitats for *A. maxima* [150].

Recently, there has been a rise in interest in *A. maxima*’s commercial cultivation. This happens most often in open ponds where lighting and temperature can be left uncontrolled. But in addition to these advantages, there are also several disadvantages, like low biomass productivity [155] because of pollution and the reservoir’s regular water evaporation [156]. Growing the algae in photobioreactors results in a higher quality product and a rise in biomass [157], where pollutants are less prevalent and the amount of carbon dioxide and light flux is regulated. The chemical makeup of the harvested algae as well as their productivity and biomass depend on the bioreactors’ temperature conditions being controlled. Regarding the application of Spirulina species in the food, pharmaceutical, and medical industries, the latter is especially important. Research shows that the highest amount of µ-linolenic acid was produced at the optimal growth temperatures of 35 and 40 °C for *A. maxima* [158]. Temperature and solar radiation have a direct impact on Spirulina spp. biomass productivity as well as its enhanced nutritional qualities: elevated levels of protein, phycocyanin, and polyunsaturated fatty acids, primarily γ-linolenic [159].

#### Chemical composition

Bioactive metabolites can be effectively produced by spirulina [160, 161]. Over 60% of the dry bulk is made up of their protein content [162]. In addition to being a noteworthy source of proteins, Spirulina also contains large amounts of minerals (6.9% w/w), crude fibers (8.5%), carbohydrates (10.3% w/w), and lipids (7.2% w/w) [161]. According to certain writers, it contains sterols, γ-linolenic acid (GLA), polyunsaturated fatty acids, and necessary amino acids [163]. Vitamins B3, B6, B12, and K are the most abundant in their raw material. Niacin B3 is the most abundant vitamin [164]. It was demonstrated that the diversity in the β-carotene content in *Arthrospira* was caused by significant changes in the harvest season, geographical region, and drying method [165].

Algal lipids’ fatty acid composition was primarily composed of palmitic and linoleic acids, with smaller amounts of myristic, γ-linolenic, palmitoleic, and oleic acids [164]. An examination of the amino acid composition shows that Spirulina is unique in that it contains high concentrations of certain amino acids, such as glutamate, aspartate, alanine, leucine, arginine, valine, glycine, tyrosine, and proline [166].

Algal powder contains a high level of minerals, with the highest overall amounts found in potassium, salt, phosphorus, calcium, magnesium, and iron [151]. Additionally, it was discovered that cyanobacterial selenium, which exists as selenite and selenomethionine, is abundant in the algae [167]. *Arthrospira* is widely regarded as a superior source for phycocyanin (CPC) synthesis [168, 169].

#### The uses and the antimicrobial potential of Spirulina

A major factor in its approval by the FDA and sale as a food supplement is the dry mass’s 65% protein content. The protein found in Spirulina contains all of the essential amino acids; in contrast, *A. platensis* contains 10% w/w carbohydrates and 7% w/w lipids, of which 1.5-2% are polyunsaturated fatty acids [170]. Because of its demonstrated in vitro and in vivo bioactivities, *Arthrospira* is a useful tool for complementary and alternative medicine. It also has a lot of potential for use in conventional medicine [171, 172].

Its antioxidant, antiviral, antibacterial, anti-inflammatory, immunostimulant, and antineoplastic qualities are extensive [171]. In addition to having antidiabetic, anti-obesity, and metalloprotective hepatoprotective qualities, some allergies and rhinitis can be prevented by consuming Spirulina. Additionally, it has been suggested that it affects hyperglycemia, hyperlipidemia, anemia, cardiovascular illnesses, and neurodegenerative disorders. The bioactive substances in *Arthrospira* are thought to be responsible for these actions either singly or in combination.

Phenol compounds, β-carotene, α-tocopherol (vitamin E), calcium spirulan, phycocyanin, caffeic and chlorogenic acid, as well as vitamins and minerals, are all plentiful in *Arthrospira*. It also contains a lot of fatty acids, especially important PUFAs like omega-3 and omega-6 [171].

Free fatty acids (FFAs) are recognized to have a broad antimicrobial action [173]. Fatty acids, in particular, polyunsaturated fatty acids (PUFAs), are among the most abundant and effective antibacterial compounds found in microalgae, possessing untapped therapeutic potential [174]. The double bonds found in unsaturated fatty acids are likely to contribute to their higher antibacterial effect when compared to saturated fatty acids [175]. Their mode of action is mostly due to their surfactant characteristics, which tear down cell membranes and allow electrons to seep out. Additionally, they cause peroxidative processes and block the synthesis of ATP, other bacterial enzymes, and electron transport systems [176, 177]. Phycocyanin, phycocyanobilin, and allophycocyanin are three more important antimicrobial (antibacterial, antifungal, and antiviral) chemicals found in *Arthrospira* that also have some anticancer properties [178].

In addition, the taxon contains terpenes, flavonoids, and other phenolic chemicals that have demonstrated their antibacterial qualities [178]. Prolonged exposure to high concentrations of phenolic compounds can denature proteins in bacterial cell walls and membranes by creating hydrogen bonds that damage the proteins and cause leakage [176, 179]. They probably affect enzymes at low doses, particularly those that produce energy [180]. The impact on spore germination and binding and damage to lipids in cell membranes and organelles could be the cause of the antifungal action [181]. Terpenoids can damage the porins in bacterial cell walls and impede the flow of nutrients by forming a strong link polymer [182]. Alkaloids can disrupt peptidoglycan components in bacterial cell walls, resulting in wall damage and eventual bacterial cell death. The bacterial cell wall voltage can be lowered by detergents known as saponins, which permeabilize the membrane. To do this, they lower the surface tension of the cell wall, which lets them in, messes with the metabolism of the cell, and eventually kills the bacteria. Quinone compounds can bind to cell proteins and produce malfunctions that can disrupt the metabolism of the cell. Microalgal steroids break down the membrane of the bacterium [182].

The antibacterial activity of Spirulina’s water extract was tested in vitro for its effect against *Staphylococcus aureus*, *E. coli*, and *Klebsiella*, using the paper disc diffusion method. Minimal inhibitory effects were shown by *Klebsiella*, while *E. coli* and *S. aureus* were the most susceptible strains to Spirulina’s water extract [183]. Another study involved the testing of Spirulina’s aqueous extract for antibacterial activity against *P. aeruginosa*, *K. pneumoniae*, *E. coli*, *S. aureus*, and *S. pneumoniae* that were isolated from different infection sources. The results showed that Spirulina’s aqueous extract had inhibition activity against these tested bacterial isolates [184]. However, It was reported that no activity of C-phycocyanin has been observed against *A. baumannii* [185]. Table 2 shows previous studies reporting the antibacterial activity of *A. maxima*.

**Table 2:** Studies revealing the antibacterial activity of *A. maxima*

Investigation	Outcome	Reference
Application of *Arthrospira* for medicinal purposes and in the food industry.	There is evidence that *Arthrospira* has antibacterial and antifungal activity.	[171]
Investigating purified C-phycocyanin from *Spirulina platensis* against several bacteria such as *K. pneumoniae.*	Phycocyanin extracted and purified from the algae can be used to combat drug resistance, as it significantly inhibits drug-resistant *K. pneumoniae.*	[185]
Assessment of antibacterial activity of Spirulina’s extract against some bacteria such as *S. aureus*, *E. coli*, and *Klebsiella.*	Minimal inhibitory effects were shown by *Klebsiella*, while *E. coli* and *S. aureus* were the most susceptible strains to Spirulina’s water extract.	[183]
testing of Spirulina’s aqueous extract for antibacterial activity against *P. aeruginosa*, *K. pneumoniae*, *E. coli*, *S. aureus*, and *S. pneumoniae.*	Spirulina’s aqueous extract had inhibition activity against *P. aeruginosa*, *K. pneumoniae*, *E. coli*, *S. aureus*, and *S. pneumoniae.*	[184]

### ZnO nanoparticles (ZnO-NPs)

In contemporary materials science, nanotechnology is a prominent topic for research. Advanced medical operations, food processing, novel fabric components, and agricultural production are just a few of the unique uses for this technology [186, 187]. It is generally believed to refer to the synthesis, characterization, and study of materials at the nanoscale (1–100 nm). This nanoscale dimension frequently results in nanoparticles having larger surface areas than macro-sized particles [188, 189].

Many studies have looked at the various geometries and remarkable antibacterial activity of nano-sized ZnO-NPs versus several species of bacteria [190–192]. ZnO-NPs are currently being investigated as an antibacterial agent in formulations at the microscale and nanoscales. ZnO-NPs exhibit significant antibacterial activity when reduced to the nanoscale. The ZnO-NPs can then engage with the bacterial core or surface when they enter the cell, exhibiting distinct bactericidal processes as a result [193, 194]. These unusual compounds’ largely toxic interactions with microorganisms have been used for antibacterial purposes, including in the food sector. Numerous investigations have reported that ZnO-NPs are not harmful to human cells [195, 196]. Because of their high degree of biocompatibility with human cells and toxicity to microbes, this trait makes them effective as antibacterial agents [197]. The many antibacterial actions of ZnO-NPs are mainly attributed to their high specific surface area-to-volume ratios [198], as well as the unique physicochemical characteristics.

The ZnO-NPs are mainly synthesized either chemically or biologically. Some benefits of using biological resources to create NPs include low energy usage, moderate technology, and the absence of hazardous chemicals [199]. The process of producing nanoparticles with the aid of microorganisms or plants is referred to as “biosynthesis”. These “green synthesis” nanoparticles have been applied to medication delivery, gene therapy, and a range of medical conditions, among their properties are those of an antioxidant, biofilm inhibitor, antibacterial, anti-inflammatory, and anticancer [200]. Green ZnO-NPs have gained a recent concern as a new approach as antimicrobial agents because they are more affordable, durable, and stable than other nanoparticles that are produced using traditional techniques [201]. Because eukaryotic organisms like fungi can create a lot of enzymes, oxide nanoparticles made from them are advantageous [202]. Freshwater and marine microorganisms known as microalgae are sustainable, cost-effective, and renewable. They can produce important bio-components such as phenols, tannins, alkaloids, sterols, saponins, flavonoids, and tocopherols. These substances contribute to the creation of green ZnO-NPs by reducing, chelating, and capping [203]. When the algal extract is added dropwise with continuous stirring to zinc acetate, these biomolecules can reduce the Zn ions to zinc oxide which precipitates as a powder that is collected and dried [25].

As a major health concern that has attracted attention from people all over the world, bacterial infectious illnesses are seen as a burden to public health. Therefore, there is an increasing need to create innovative antibacterial medicines against strains of bacteria, primarily significant food pathogens such as *K. pneumoniae* and *A. baumannii*.

#### Application prospects of ZnO-NPs

ZnO-NPs are thought to be a somewhat safe metal oxide that has antibacterial, antifungal, and wound-healing properties in addition to the intrinsic capacity to produce reactive oxygen species (ROS) and cause apoptosis. ZnO-NPs’ superior antibacterial capabilities and Zn^+ 2^’s epithelial-stimulating effect have been effectively utilized in wound dressings. ZnO-NPs exhibit strong antifungal action in addition to their outstanding antibacterial activity. It has been established that ZnO-NPs can dramatically reduce penicillium and mucor growth and reproduction and that ZnO-NP morphologies are crucial to the inhibitory effect [204].

#### Antibacterial activity of ZnO-NPs

Benefits of ZnO-NPs include the following: powerful antibacterial activity against a range of infections at low doses (0.16–5.00 mmol/L), quite reasonably priced [205]. There have been numerous attempts to assess and investigate ZnO-NPs’ antibacterial activities. Researchers examined the antibacterial properties of ZnO-NPs made with pomegranate leaf aqueous extracts in October 2020. They discovered that ZnO-NPs worked well against all of the pathogenic strains they had chosen, including *K. pneumoniae*. They claimed that both ZnO-NPs are useful substitute antibacterial agents [206]. ZnO-NPs have been shown in another investigation to exhibit potent antibacterial activity and to enhance the antibacterial activity against *E. coli* and *K. pneumoniae* [207].

A prior study found that the generation of various active oxygen species (ROS), including hydrogen peroxide, superoxide radicals, hydroxyl radicals, and singlet oxygen, is likely responsible for the antibacterial efficacy of ZnO-NPs. Studies have demonstrated that ROS can disrupt biological processes by influencing biomolecules like proteins, lipids, and nucleic acids [208, 209].

When tested against carbapenem-resistant *K. pneumoniae*, biosynthesized ZnO-NPs using *Aspergillus niger* revealed an inhibitory effect against tested *K. pneumoniae*. Moreover, they accelerated the healing of infected wounds [210]. Another study was conducted to investigate the effects of MIC and sub-MIC levels of ZnO NPs on the biofilm formation of *K. pneumoniae* and *S. aureus*. It was found that when treating the organisms with sub-MIC levels of ZnO NPs, an enhanced biofilm formation occured when compared with the untreated sample. Moreover, no microbial growth was observed for the samples treated with the MIC level of ZnO NPs hence proved to be an effective antibacterial [211].

Regarding the effect of ZnO-NPs on *A. baumannii*, it was reported that all of the chemically and biologically synthesized ZnO-NPs showed activity against the RS307 strain of *A. baumannii* at different levels [212, 213]. Moreover, combination of ZnO-NPs with ciprofloxacin and ceftazidime against multidrug-resistant *Acinetobacter baumannii* resulted in increasing the antibacterial activities of both antibiotics in the presence of a subinhibitory concentration of ZnO-NPs. It also increased the uptake of antibiotics and changed the bacterial cells from rod to cocci forms which indicate that ZnO-NPs may potentiate the antimicrobial action of ciprofloxacin and ceftazidime [214]. A previous study conducted in Saudi Arabia reported that ZnO-NPs had antibacterial activity with a MIC value of 2000 µg/mL against *A. baumannii* [215]. The ZnO-NPs were reported to inhibit the growth of *A. baumannii* after 120 min at a concentration of 5000 µg/ml according to a recent study [216]. Another study aimed to investigate the effect of ZnO-NPs on the expression of genes involved in toxin-antitoxin (TA) systems in multidrug-resistant *A. baumannii* [217]. It reported a significant correlation between the presence of HicA/HicB (TA) system and resistance to ceftazidime, meropenem, imipenem, and cefepime (*p* < 0.05), and the presence of HipA/HipB (TA) system and resistance to ceftazidime, meropenem, imipenem, and cefepime (*p* < 0.05). In the presence of ZnO-NPs, the expression of all investigated genes was reduced. Table 3 reveals the recent studies elucidating the antibacterial action of ZnO-NPs.

**Table 3:** Studies revealing the antibacterial activity of ZnO-NPs

Investigation	Outcome	Reference
Testing biosynthesized ZnO-NPs against carbapenem-resistant *K. pneumoniae*	Green ZnO-NPs showed an antibacterial against tested *K. pneumoniae* and reduced wound healing period	[210]
Testing biologically prepared ZnO-NPs using leaves and flowers of pomegranate against *K. pneumoniae.*	Both prepared ZnO-NPs demonstrated antibacterial activity against tested *K. pneumoniae.*	[206]
Investigating the efficacy of ZnO-NPs against *E. coli* and *K. pneumoniae.*	ZnO-NPs revealed a significant antibacterial activity against both tested strains.	[207]
Investigated the effects of MIC and sub-MIC levels of ZnO-NPs on the biofilm formation of *K. pneumoniae* and *S. aureus.*	No microbial growth was observed when treated with MIC level of ZnO-NPs.	[211]
Investigating Chemically and biologically synthesized ZnO-NPs against *A. baumannii.*	Both ZnO-NPs inhibited the growth of the RS307 strain of *A. baumannii* at different levels.	[212]
Studying the efficacy of the combination of ZnO-NPs with ciprofloxacin and ceftazidime against *A. baumannii.*	The combination resulted in increasing the antibacterial activities of both antibiotics in the presence of a subinhibitory concentration of ZnO-NPs, increased the uptake of antibiotics, and changed the bacterial cells from rod to cocci forms.	[214]
Determination of synergistic effects of antibiotics and Zno-NPs against isolated *E. Coli* and *A. Baumannii.*	ZnO-NPs had antibacterial activity with a MIC value of 2000 µg/mL against *A. baumannii.*	[215]
Investigating the MIC of ZnO-NPS against biofilm-forming *A. baumannii*	ZnO-NPs could inhibit growth at 5000 µg/ml.	[216]
Studying the impact of ZnO-NPs on the expression of the genes involved in toxin-antitoxin systems in MDR *A. baumannii.*	In the presence of ZnO-NPs, the expression of all investigated genes was reduced.	[217]

#### Mechanisms of antibacterial activity of ZnO-NPs

**Fig. 4:**
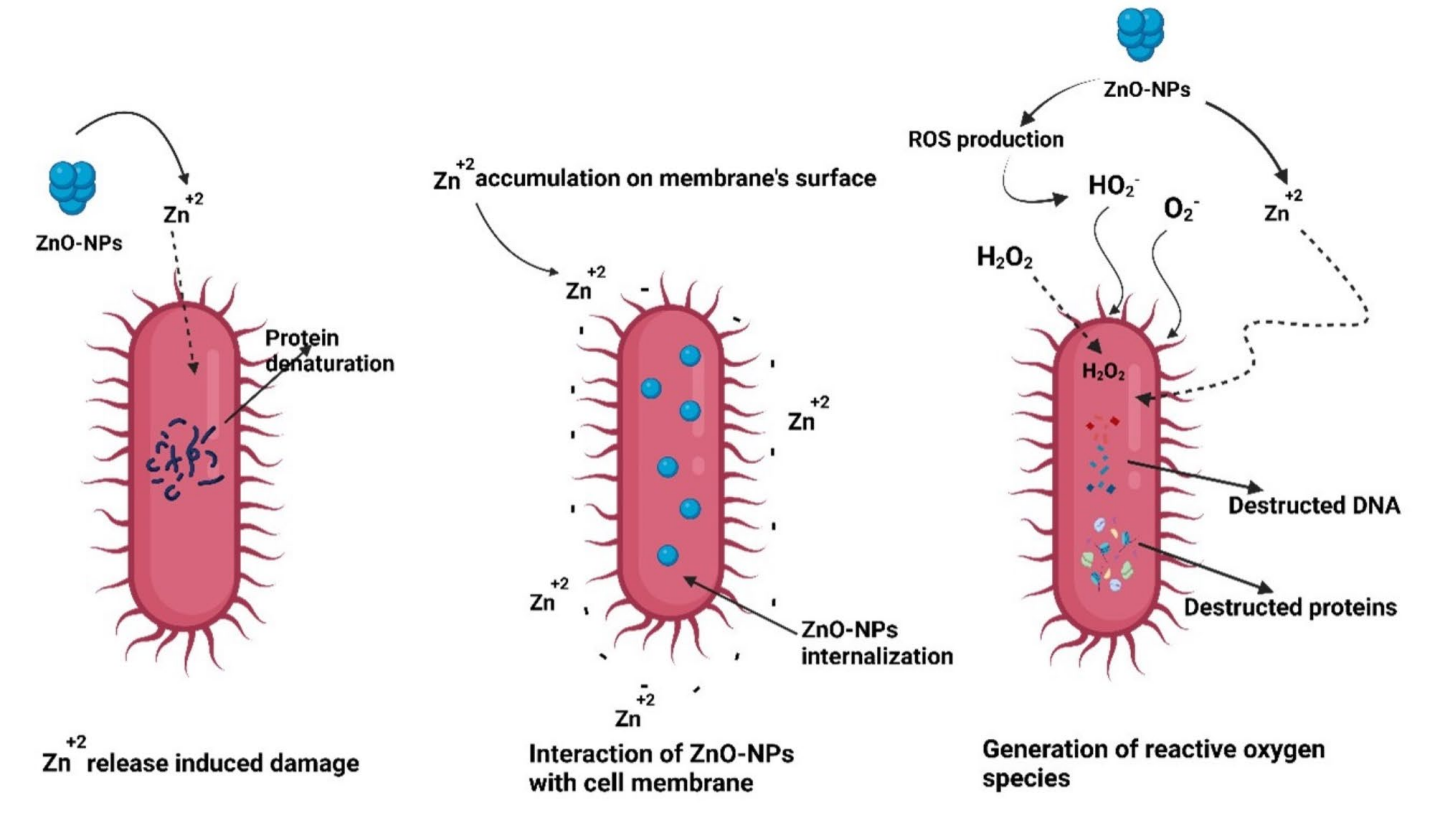
Mechanism of antibacterial activity of ZnO-NPs

Different hypothesized processes underlie ZnO-NPs’ antibacterial action. As shown in Fig. 4, they include the production of ROS, damage caused by the release of Zn^+ 2^, and cell membrane with ZnO-NPs interaction [218–224].

##### Generation of reactive oxygen species (ROS)

The creation of ROS is the most widely accepted and popular mechanism for ZnO-NPs’ antibacterial effect. To have a bactericidal impact, ROS can break the chemical connections that hold bacteria’s organic matter together. Among these are the following: H_2_O_2_ can permeate the cell membrane, damaging it and rupturing DNA and protein within, which has a bactericidal effect. Because negatively charged peroxide cannot flow through a cell membrane, OH^−^ accumulates on the surface of bacterial cell membranes, rupturing the membrane [218].

##### Zn^+ 2^ Release induced damage

According to the hypothesis, ZnO-NPs in an aqueous solution can release Zn^+ 2^ gradually. Zn^+ 2^ can pass through cell membranes, denaturing proteins and preventing cell division. Moreover, the electron transport system can be damaged by Zn^+ 2^ which might result in a respiratory disease that affects cells. Through interaction with the active site of the enzyme, the released Zn^+ 2^ ions can block β-lactamase enzymes, preventing them from hydrolyzing β-lactam drugs. Direct metal ion contact can disrupt enzyme function, akin to the mechanism of action of certain β-lactamase inhibitors such as clavulanic acid [225]. A study that looked at oxygen vacancies’ frequency in the dark state and also investigated the antibacterial effects of different ZnO-NPs of different particle sizes discovered that the main antibacterial agents were ZnO-NPs and the cell surface- adsorbed Zn^+ 2^ [221]. According to certain research, Zn^+ 2^ is electrostatically drawn to the bacterial cell membrane surface which is negatively charged, disrupting the balance of charge there and severely deforming the cell before ultimately causing lysis of bacteria [219]. They were studied to determine whether ZnO-NPs damaged the *E. coli* cell membrane. Additional investigation revealed that cell membranes and ZnO-NPs may have directly impacted the damage [220].

##### Interaction of ZnO-NPs with the cell membrane

According to certain theories, ZnO-NPs interact with bacteria and subsequently degrade their surface to provide their antibacterial effects. This mostly consists of membrane malfunction caused by the accumulation of positively charged Zn^+ 2^ on the surface of the cell membrane and disturbance of bacterial substance energy metabolism caused by the internalization of ZnO-NPs. The process by which particles smaller than 10 nm can pass through the cell plasma membrane is known as particle internalization [218]. ZnO-NPs can enter the cytoplasm as well. Additionally, ZnO-NPs and bacterial cell membranes may interact to make the membranes more permeable. Once the ZnO-NPs are absorbed by the cells, they will limit or eliminate the metabolic exchange of materials and energy between the bacteria and their environment, which will cause the bacteria to die [220].

### Future perspectives

Spirulina, a nutrient-dense blue-green algae, demonstrates considerable potential as an antibacterial agent due to its bioactive compounds like phycocyanin and polysaccharides, which can disrupt bacterial cell walls and inhibit growth. Its anti-inflammatory and antioxidant properties may also contribute to its antibacterial effects and decrease the opportunity for bacterial contamination. Future research should focus on elucidating these mechanisms, optimizing pharmaceutical formulations for stability and bioavailability, and exploring potential synergistic effects with other antimicrobial agents.

Green ZnO-NPs, produced through eco-friendly methods, have a large surface area and can produce ROS, which harm bacterial cells, making them excellent candidates for use as antibacterial agents. Future research should focus on optimizing their size, shape, and stability, and understanding their precise antibacterial mechanisms. Comprehensive preclinical and clinical studies are essential to evaluate their safety and efficacy, with structured trials needed to confirm their therapeutic benefits in humans. Addressing challenges related to standardization and regulatory approval will be key to integrating Spirulina and green ZnO-NPs into mainstream treatments as a sustainable alternative to traditional antibiotics.

## Conclusion

To sum up, research is needed to validate the potential activity of both *A. maxima* and ZnO-NPs as antibacterial agents against infections caused by bacteria resistant to carbapenems. Therefore, both can be viewed as essential additions to the next research to fight diseases caused by bacteria resistant to numerous medications.

## Data Availability

No datasets were generated or analysed during the current study.
